# Development and Validation of a New Storage Procedure to Extend the In-Use Stability of Azacitidine in Pharmaceutical Formulations

**DOI:** 10.3390/ph14090943

**Published:** 2021-09-21

**Authors:** Antonella Iudicello, Filippo Genovese, Valentina Strusi, Massimo Dominici, Barbara Ruozi

**Affiliations:** 1 Pharmaceutical Department, Azienda USL of Modena, Largo del Pozzo 71, 41121 Modena, Italy; 2 Nuclear Medicine Unit, Oncology and Hematology Department, Azienda Ospedaliero-Universitaria of Modena, Largo del Pozzo 71, 41124 Modena, Italy; 3 Centro Interdipartimentale Grandi Strumenti, University of Modena and Reggio Emilia, Via Campi 213/A, 41125 Modena, Italy; fgenovese@unimore.it; 4 Scientific and Technological Park of Medicine “Mario Veronesi”, Via 29 Maggio 6, 41037 Mirandola, Italy; valentina.strusi@tpm.bio (V.S.); mdominici@unimore.it (M.D.); 5 Division of Medical Oncology, Department of Medical and Surgical Sciences for Children & Adults, University of Modena and Reggio Emilia, Hospital of Modena, Largo del Pozzo 71, 44125 Modena, Italy; 6 Department of Life Sciences, University of Modena and Reggio Emilia, Via Campi 213/A, 41125 Modena, Italy; barbara.ruozi@unimore.it

**Keywords:** anticancer drugs, azacitidine, drug degradation, limits of use, practical stability, in-use stability

## Abstract

Stability studies performed by the pharmaceutical industry are principally designed to fulfill licensing requirements. Thus, post-dilution or post-reconstitution stability data are frequently limited to 24 h only for bacteriological reasons, regardless of the true physicochemical stability which could, in many cases, be longer. In practice, the pharmacy-based centralized preparation may require preparation in advance for administration, for example, on weekends, holidays, or in general when pharmacies may be closed. We report an innovative strategy for storing resuspended solutions of azacitidine, a well-known chemotherapic agent, for which the manufacturer lists maximum stability of 22 h. By placing the syringe with the azacitidine reconstituted suspension between two refrigerant gel packs and storing it at 4 °C, we found that the concentration of azacitidine remained above 98% of the initial concentration for 48 h, and no change in color nor the physicochemical properties of the suspension were observed throughout the study period. The physicochemical and microbiological properties were evaluated by HPLC–UV and UHPLC-HRMS analysis, FTIR spectroscopy, pH determination, visual and subvisual examination, and sterility assay. The HPLC-UV method used for evaluating the chemical stability of azacitidine was validated according to ICH. Precise control of storage temperature was obtained by a digital data logger. Our study indicates that by changing the storage procedure of azacitidine reconstituted suspension, the usage window of the drug can be significantly extended to a time frame that better copes with its use in the clinical environment.

## 1. Introduction

Many of the drugs used in modern medicine are licensed with very limited stability data, which often are not enough to fulfill the ways of drugs being handled in the clinical environment.

Usually, the stability limit given by the pharmaceutical industry is principally based on the possible risk of biological contamination and not on the real physicochemical stability. However, nowadays, in most hospitals, the reconstitution and preparation of drugs, especially for anticancer ones, takes place in centralized compounding units under good hospital pharmacy manufacturing practice, in which the principles of good manufacturing practice (GMP) were applied to hospital pharmacy compounding [1].

Ideally, drug development studies of the pharmaceutical industry should generate enough stability data to allow for a more flexible clinical application, that should be available to the community of pharmacists beyond the official package insert. Unfortunately, full access to stability experiments supplied by manufacturers to registering authorities is not available, as for other data obtained during preclinical experiments or clinical trials [2]. Therefore, sometimes there is the need to establish a range of validated assays testing different ways of preparing and storing drugs for longer periods, extending the stability limits indicated in package inserts or in the summary of product characteristics (SPC) to take into account practical needs [1]. The purpose of these assays is to establish the in-use stability of a drug, that is the period during which the product can be used, after the first opening, retaining the quality within an accepted specification [3].

When reconstitution and dilution are carried out in a sterile environment following the United States Pharmacopeia Chapter’s <797> recommendations, it could be reasonable to extend the expiring dates of drugs from 24 h to 10 or even 14 days, providing there are no stability or physicochemical issues with the product [4].

Azacitidine (4-amino-1-β-D-ribofuranosyl-1,3,5-triazin-2(1H)-one) is an antimetabolite pyrimidine nucleoside analog, supplied as Vidaza^®^ (Celgene, Italy). Due to its demethylating properties, it is able to affect the expression of genes controlling cell growth and differentiation and, therefore, azacitidine is authorized by the European Medicines Agency (EMA) for the treatment of various myelodysplastic syndromes.

Each vial of Vidaza^®^ contains azacitidine and mannitol (each 100 mg) as a sterile lyophilized powder, that must be resuspended in 4 mL of sterile water for injection to produce a suspension for subcutaneous injection.

According to SPC, the product, before reconstitution, can be stored for up to 4 years. Differently, the 25 mg/mL reconstituted suspension can be kept for 45 min at room temperature (25 °C) or 8 h at 2–8 °C after reconstitution with sterile water for injection, not previously refrigerated. Instead, when refrigerated sterile water for injection (2–8 °C) is used, the suspension remains stable for another 22 h if stored at 2–8 °C [5]. Indeed, the azacitidine is very unstable in an aqueous solution [6,7,8,9] and it is rapidly hydrolyzed to several degradation products (DPs) in a temperature-dependent process [10,11].

The 22 h stability does not allow the Vidaza^®^ preparation in advance, which could be very important especially for the weekend because Vidaza^®^ (25 mg/mL) suspensions have to be subcutaneously administered for seven consecutive days of a 28-day cycle.

The short stability of the Vidaza^®^ suspensions reported on SPC requires that the suspensions must be prepared daily, including on weekends, holidays, or in general when pharmacies may be closed.

To bypass the stability issue, many institutions use alternative dosing schedules such as a 5-day regimen or a 7-day regimen with an interruption over the weekend. However, there is evidence that the 7-day regimen may be associated with better patient response [12,13,14,15,16].

Different studies, selected by using the Stabilis^®^ database [17] (accessed on 5 May 2021), showed that the solubility, as well as the degradation of azacitidine in an aqueous medium, are temperature-dependent: the higher is the temperature, the higher is the solubility, but cold temperatures slow down the degradation process and can theoretically help to prolong the shelf life of reconstituted Vidaza^®^ compared with that indicated by the manufacturer. However, published results are not that recent, heterogeneous in terms of quality and relevance, and many of them do not take practical needs into account and the results need to be confirmed using the formulation currently administered in clinical practice (i.e., a 25 mg/mL aqueous azacitidine suspension) [18,19]. Moreover, most of the studies considered only chemical stability and did not evaluate a potential variation over time of the physical and microbiological parameters of reconstituted Vidaza^®^ [7,10,11,12,20,21].

Given that clinical needs may deviate from licensing requirements, the first aim of this study was to determine the practical stability (or in-use stability) of Vidaza^®^ (25 mg/mL) suspension, reconstituted according to routine clinical operating conditions (using standardized procedures) with refrigerated sterile water for injection (2–8 °C) and stored at 2–8 °C, as reported in the SPC. The second aim was to evaluate the possibility of preparing syringes of azacitidine suspension in advance, which can be administered one or two days later when the centralized compounding unit of reconstitution and preparation of anticancer drugs may be closed, or in case of administration cancellation or postponing [22].

## 2. Results

### 2.1. HPLC–UV Analysis

It is known that rapid and reversible hydrolysis of the azacitidine occurs in an aqueous medium, which leads to N-formyl RibosylGuanylUrea (RGU-CHO) formation, which is irreversibly hydrolyzed into RibosylGuanylUrea (RGU). It is a two-stage degradation [7,8,9,10,11,23]. These hydrolysis products do not produce toxicological or therapeutic effects, but they lead only to decreasing azacitidine potency [9,10,24].

**Figure 1:**
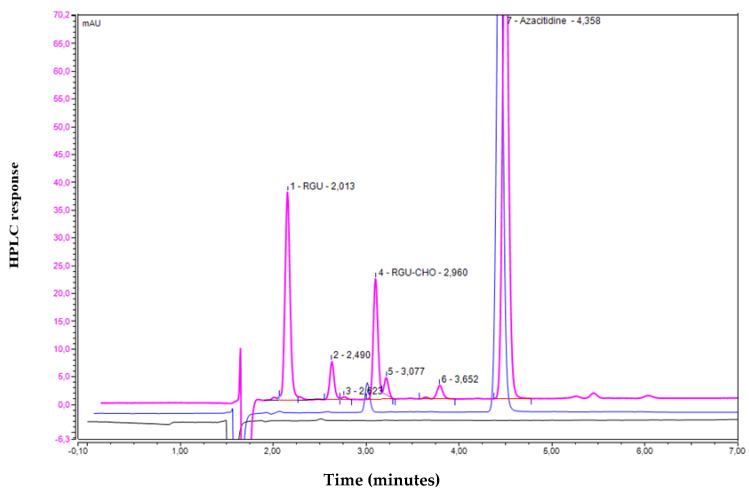
Chromatogram showing the peak related to water for injection (black line); chromatogram showing the peak related to azacitidine (50 µg/mL) standard solution freshly prepared (blue line); chromatogram of a Vidaza^®^ (25 mg/mL) preparation subjected to heat stress (violet line). No interfering materials were detected.

However, already at time zero, chromatograms showed, besides the azacitidine peak with an average retention time (RT) of 4.3 min, the presence of four other peaks, including two peaks attributed to RGU-CHO and RGU with an average RT of 2.9 min and 2.0 min, respectively [7,8,9,10,11,23], and other two peaks with an average RT of 2.5 min and 3.6 min, which were more clearly detected in chromatograms obtained from Vidaza^®^ (50 µg/mL) subjected to heat stress (see the violet line, Figure 1).

**Figure 2:**
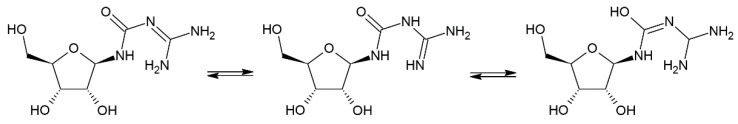
Three RGU tautomeric forms.

**Figure 3:**
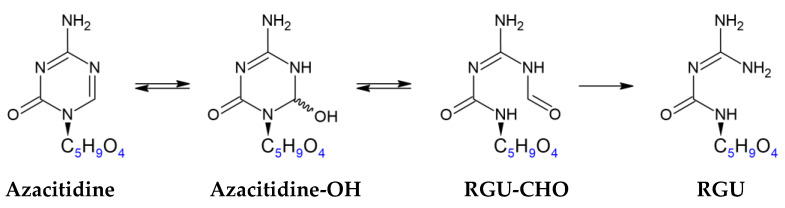
Path for 5-azacytidine hydrolysis proposed by Notari and Chan with carbinolamine intermediate formation, which is slow at neutral pH.

These two peaks were attributed by UHPLC-HRMS analysis to RGU tautomeric forms (Figure 2) and a carbinolamine intermediate (Figure 3), respectively, as supported by the works of Notari et al. and Chan et al. [8,9].

Over time, the areas under the curve (AUC) of RGU tautomeric forms, RGU-CHO, and carbinolamine intermediate remained relatively stable: these DPs formed immediately, and then their AUC remained relatively stable.

**Figure 4:**
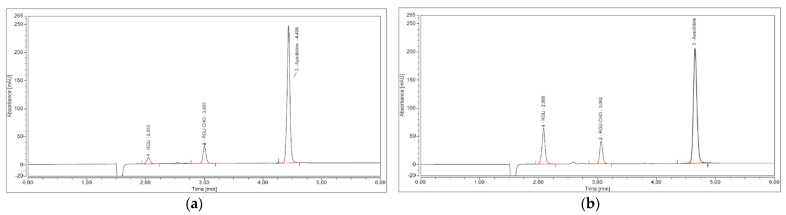
Comparison of chromatograms between the day the Vidaza^®^ (25 mg/mL) was reconstituted (**a**) and the 96 h after the reconstitution (**b**): we can observe a decrease of the azacitidine peak that favors an increase of the RGU peak.

In contrast, a symmetrical evolution of the AUC was observed between azacitidine and RGU: the RGU formed more slowly, then its rate of increase accelerates (Figure 4).

### 2.2. Forced Degradation

Figure 1 shows the chromatograms of the diluent (black line), a 50 µg/mL azacitidine standard solution freshly prepared (blue line), and one sample of Vidaza^®^ (50 µg/mL) subjected to heat stress (violet line).

After 12 h at 50 °C/43% RH, no formation of other peaks was observed.

No co-eluting peaks were generated from stress conditions of heat, indicating the specificity and the suitability of the chromatographic method for use as a stability-indicating assay (see Supplementary Materials).

### 2.3. UHPLC-HRMS Analysis

The mass spectrometric study, performed with a similar setup to the one adopted for HPLC-UV analyses, allowed the identification of the DPs with an average RT of 2.5 min and 3.6 min.

**Figure 5:**
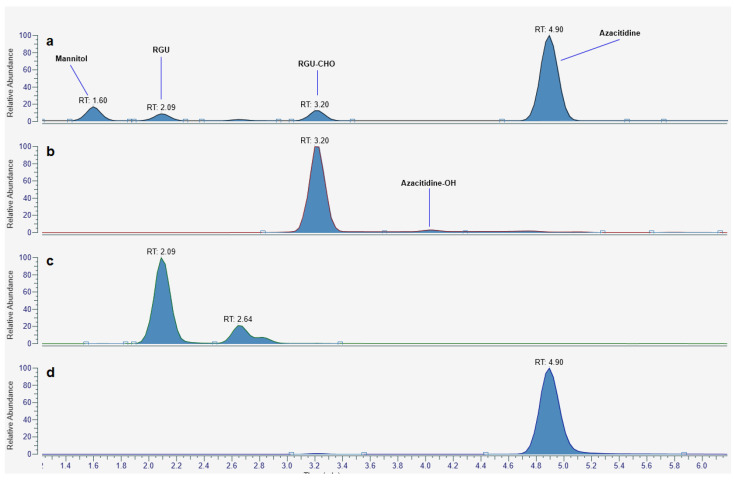
Typical ESI+ base peak chromatogram of a partially degraded azacitidine sample (**a**); extracted ion chromatogram (*m*/*z*: 263.0986, corresponding to the protonated form of RGU-CHO), showing an additional peak at RT = 4′, attributable to the hydrated form of azacitidine or an RGU-CHO tautomer (**b**); extracted ion chromatogram (*m*/*z*: 235.1037, corresponding to the protonated form of RGU), showing two more peaks attributable to the two RGU tautomers at RT = 2.6′ (**c**); extracted ion chromatogram (*m*/*z*: 245.0881, corresponding to the protonated form of azacitidine) (**d**).

The hypothesized intermediate structures for the 5-aza degradation were all confirmed by UHPLC high-resolution mass spectrometry. All the recorded ESI+ MS spectra of the intermediates were consistent with the proposed structures, both in terms of mass shift of the monoisotopic parent ion (<1 ppm) and isotopomers pattern. Interestingly, extracted ion chromatograms of the species of interest revealed that other chemical entities, which we attributed to RGU/RGU-CHO tautomers or the intermediate hydrated azacitidine, were present in the sample (Figure 5). HR mass spectra of each species are provided as Supplementary Materials.

### 2.4. Chemical Stability

The HPLC-UV analysis results were homogeneous for the three lots of Vidaza^®^ (25 mg/mL) in terms of the concentration of azacitidine at time zero (24.26 mg/mL, 24.45 mg/mL, and 24.27 mg/mL).

At time zero, the mean loss of azacitidine was 2.68%, relative to the theoretical concentration of 25 mg/mL (mean azacitidine concentration at time zero, 24.33 mg/mL), due to the immediate hydrolysis of azacitidine, which was accompanied by the formation of four degradation products (RGU tautomeric forms, RGU-CHO, carbinolamine intermediate, and RGU). These DPs were observed in all samples.

Table 1 shows azacitidine degradation relative to the mean experimental concentration at time zero (i.e., 24.33 mg/mL) at each time point after storage of reconstituted Vidaza^®^ (25 mg/mL) in three different storage conditions (A, B, and C).

By 22 h the azacitidine concentration in the original container (condition A) decreased to 23.87 mg/mL, corresponding to a mean loss of 1.86% relative to the initial experimental concentration value (24.33 mg/mL), and a mean loss of 4.52% relative to the initial theoretical concentration (25 mg/mL).

The mean loss of 1.86% was identified as the maximum acceptable change of azacitidine concentration.

**Figure 6:**
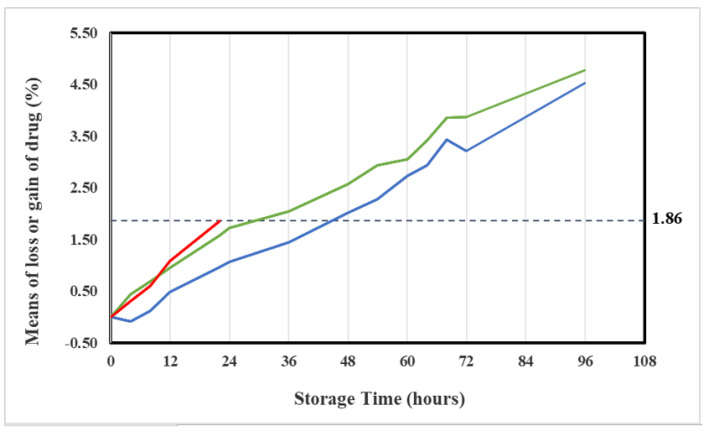
Trend of the mean percentage of azacitidine lost in the original container over 22 h (red line), in a polypropylene syringe (green line), and in a polypropylene syringe placed between two refrigerant gel packs (blue line) over 96 h. The dotted line represents the maximum 1.86% loss (relative to the initial experimental concentration) identified as the maximum acceptable change of concentration.

This mean loss of azacitidine was found out after 24 h in Vidaza^®^ suspensions stored at 2–8 °C in polypropylene syringes, and after 36 h in Vidaza^®^ suspensions stored at 2–8 °C in polypropylene syringes placed between two refrigerant gel packs (Figure 6).

Figure 6 shows that the azacitidine loss proceeds rapidly during the first 12 h (mean loss 0.94% at 12 h, see Table 1) and the rate of loss decreases thereafter. This pattern was more evident in suspensions stored in condition C (blue line) and it was in accord with the report by Hartigh et al. [10], but it was in contrast with a recent report by Legeron et al. [12].

#### 2.4.1. Infrared Spectroscopy Analysis

The information on the functional groups of a molecule is contained in its infrared (IR) spectrum.

IR spectra obtained for all samples were similar.

The specific spectral contributions from each compound were sufficiently alike (in terms of frequency). Moreover, the samples showed similar fragmentation.

**Figure 7:**
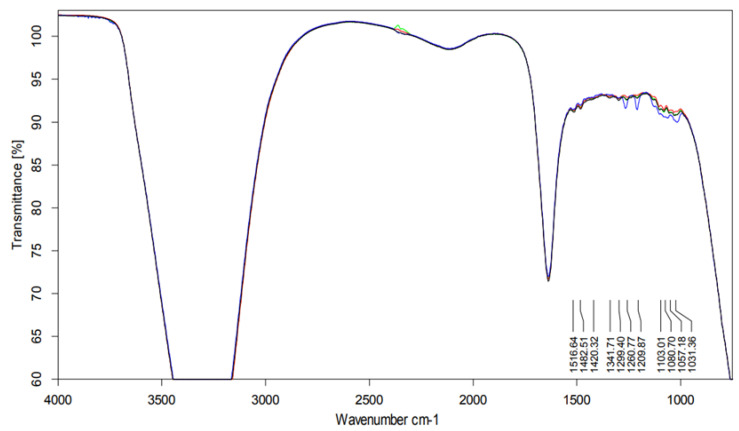
Overlap of the IR spectra referring to Vidaza^®^ (25 mg/mL) at t_0_ (blue line), stored in the original container for up to 22 h (red line), in a polypropylene syringe (black line), and in a polypropylene syringe placed between two refrigerant gel packs (green line) for up to 96 h.

In the 1200–1300 cm^−1^ region there were visible more intense bands originating most probably from the amines C–N stretch. (Figure 7).

Peak maxima for water were observed at 3450 cm^−1^ (2.898 μm), 3615 cm^−1^ (2.766 μm), and 1640 cm^−1^ (6.097 μm).

#### 2.4.2. pH Determination

The mean pH of water for injection was 6.02 ± 0.1.

At time zero, the mean pH values of three lots of reconstituted Vidaza^®^ (25 mg/mL) amounted to pH 6.8 ± 0.06. The pH slightly varied over time but remained unchanged over the observation test period (7.0 ± 0.2) (Table 2).

**Figure 8:**
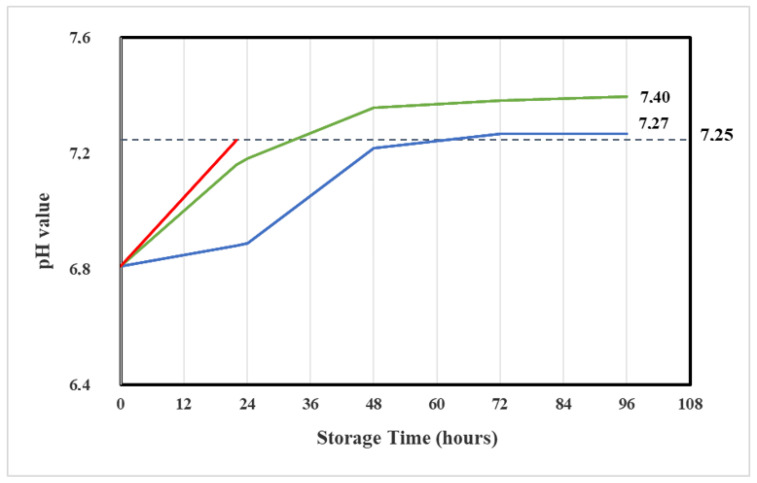
Trend of the mean pH of Vidaza^®^ (25 mg/mL) stored in the original container over 22 h (red line), in a polypropylene syringe (green line), and in a polypropylene syringe placed between two refrigerant gel packs (blue line) over 96 h. The dotted line represents the mean pH in the original container at 22 h.

The loss of the formyl group from the RGU-CHO and the formation of RGU caused a pH increase of 0.4 in reconstituted Vidaza^®^ (25 mg/mL) stored at 2–8 °C in the original container over 22 h (Table 2). The same pH increase was determined into reconstituted Vidaza^®^ (25 mg/mL) stored at 2–8 °C in a polypropylene syringe placed between two refrigerant gel packs over 48 h (Figure 8).

### 2.5. Physical Stability

#### 2.5.1. Visual Examination

On the production day, all Vidaza^®^ (25 mg/mL) suspensions were white, milky, and uniform.

In none of the test solutions were color changes, the formation of large particles, or agglomerates observed over the test period.

Over time, slight particle separations from the diluent were measured. A vigorous shaking favored the particle re-suspension.

#### 2.5.2. Subvisual Examination

##### Microscopic Observation

On the production day, the Azacitidine crystals have exhibited a needle-like shape.

**Figure 9:**
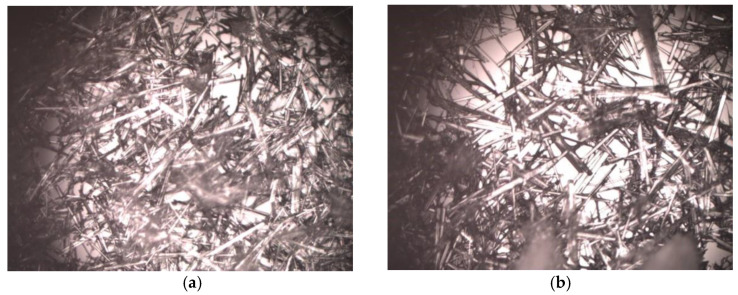
Morphology of Azacitidine crystals at time zero (**a**), and after stored Vidaza^®^ (25 mg/mL) at 2–8 °C in a polypropylene syringe for up to 96 h (**b**).

The morphology of crystals did not change under all conditions in which the Vidaza^®^ (25 mg/mL) was stored during the test period (Figure 9).

##### Particle and Size Counting

No variation in average particle diameter was observed in test solutions stored in different conditions (A, B, and C) over the test period (Table 3).

**Figure 10:**
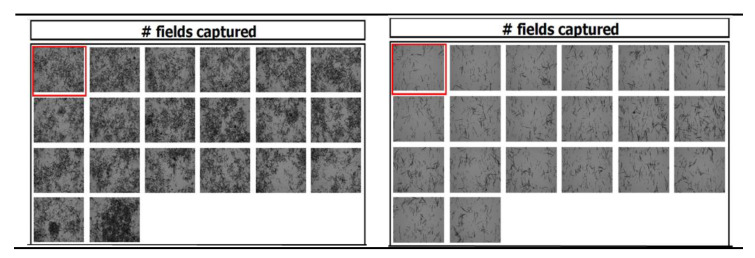
Tali^®^ image of Vidaza^®^ (25 mg/mL) at time zero (**left**), and after stored Vidaza^®^ (25 mg/mL) at 2–8 °C in a polypropylene syringe for up to 96 h (**right**).

Differently, over time the number of particles counted in each field of view of the chambers where the samples were loaded increases (Table 3), probably because of the limited capability of the Tali^®^ Image-Based cytometer for counting the adjacent or overlap particles (Figure 10, left), and because of the diminution of the number of particles per field (Figure 10, right) due to particle accumulation to edges of the chamber.

Considering the Tali^®^ Image-Based cytometer limitations and that the average particle diameter slightly varied (20 ± 2.0 µm), any sign of physical instability such as aggregation or particle microprecipitation could be excluded for up to 96 h.

### 2.6. Sterility Assay

The product sterility was maintained in syringes for up to 96 h, as well as in the original vial for up to 22 h. No growth of microbial organisms during the inoculation period was found in any samples. All samples were negative for the growth of aerobic and anaerobic micro-organisms and fungi.

No significant differences were observed among lots.

Prolonged storage in the refrigerator and into syringes did not result in microbial contamination of the content.

## 3. Discussion

The SPC of Vidaza^®^ indicates that the reconstituted drug with cold water (2–8 °C) should be kept in the original vial or drawn into a syringe and that it may then be held under refrigerated conditions (2–8 °C) for up to 22 h. After removal from refrigeration, the suspension may be allowed to equilibrate to room temperature for up to 30 min before administration. If the elapsed time is longer than 30 min, the suspension should be discarded appropriately and a new dose has to be prepared [5].

According to SPC of Vidaza^®^, our results show that there is a moderate but not significant difference in the loss of azacitidine, or generally in the evaluated physicochemical and microbiological parameters, between the Vidaza^®^ (25 mg/mL) stored refrigerated (2–8 °C) into polypropylene syringes (condition B) or in the original container (condition A) up to 22 h.

Differently, the azacitidine degrades slower if stored refrigerated (2–8 °C) between two refrigerant gel packs (condition C) because the drug degradation was observed to be very sensitive to temperature. In this storage condition, the percentage of azacitidine lost remained below 1.86% (identified as the maximum acceptable change of azacitidine concentration) throughout the first 36 h of the study and over time.

**Figure 11:**
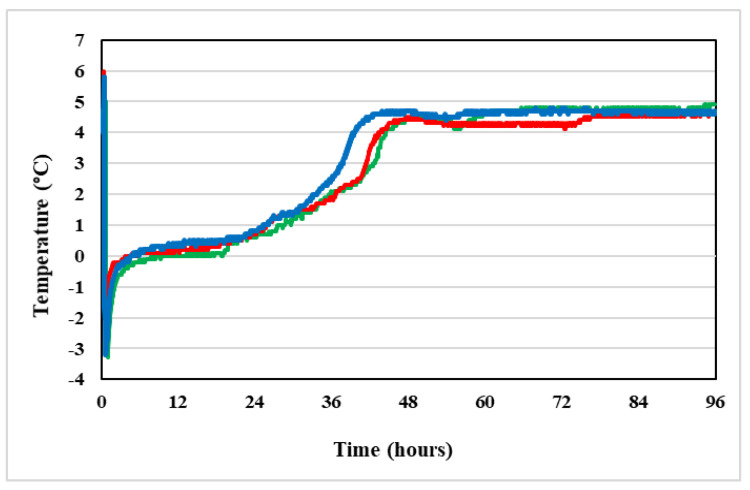
Trend of the temperature of the three Vidaza^®^ samples (red, green, and blue lines, respectively) stored refrigerated (2–8 °C) between two refrigerant gel packs, obtained placing a temperature data logger into the polypropylene syringes in contact with the drug suspension.

**Figure 12:**
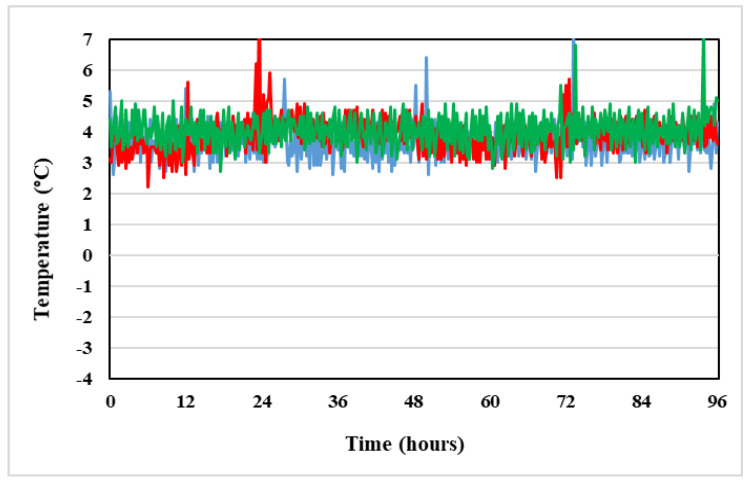
Trend of the temperature inside two refrigerators where the three Vidaza^®^ lots were placed.

This is because, during the storage of the product in condition C, the suspension temperature was registered to be from −3.0 °C to 1 °C throughout the first 24 h (Figure 11). After 24 h the refrigerant gel packs began to thaw and the temperature of the product increased up to 4.8 °C, corresponding to refrigerator temperature (Figure 12).

Two used refrigerators maintained a stable temperature within the 2 °C to 8 °C range, recommended by SPC of Vidaza^®^ (Figure 12), and so when the refrigerant gel packs thawed, the Vidaza^®^ temperature remained less than 5 °C over the whole tested period (Figure 11).

During the first 6 h of refrigerated storage between two refrigerant gel packs (condition C), the Vidaza^®^ suspension resulted as almost frozen and there was no apparent loss of azacitidine concentration (Table 1).

The use of refrigerant gel packs to increase the stability of Vidaza^®^ (25 mg/mL) suspensions is a useful alternative to freezing reported by Walker et al. and Duriez et al. [20,21] because patient care areas in hospitals and home health care settings do not always have freezers in which to store Vidaza^®^ syringes. In addition, if frozen, the product requires about 2 h for thawing [20] and pharmacists must ensure that syringes are completely thawed before drug administration [12]. Refrigeration associated with the use of refrigerant gel packs is, therefore, a more convenient alternative for storing ready-to-use Vidaza^®^ (25 mg/mL) syringes and is well suited to clinical practice.

**Figure 13:**
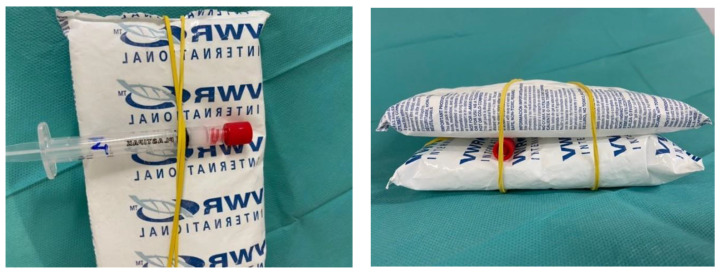
Steps to place syringe containing Vidaza^®^ suspension between two common refrigerant gel packs: step 1 (**left**) and step 2 (**right**).

The used refrigerant gel packs were commercially available. The syringes were placed between two refrigerant gel packs as shown in Figure 13 and were then stored refrigerated (2–8 °C).

Azacitidine is not included in the European or United States Pharmacopoeia [25,26]; therefore, acceptance criteria for the DPs are not available.

All studies on the stability of azacitidine in Vidaza^®^ (25 mg/mL) suspensions, available on the Stabilis^®^ website [17] (accessed on 5 May 2021), consider “acceptable” a maximum change of 5% from the initial measured concentration value according to ICH guidelines [27]. On that basis, the chemical stability of Vidaza^®^ (25 mg/mL) suspensions reconstituted with refrigerated water (2–8 °C) and stored at 2–8 °C in propylene syringes protected from light was retained to be 24 h by Walker et al., 48 h by Legeron et al., and 120 h by Vieillard et al. [12,20,28].

However, because azacitidine is very unstable in aqueous medium and already at time zero a loss of azacitidine occurs compared to the theoretical concentration of 25 mg/mL, and DPs are present, we identified the change of azacitidine concentration from its initial measured concentration value occurring at 22 h from preparation in Vidaza^®^ suspensions, reconstituted and stored refrigerated (2–8 °C) in the original container according to SPC (condition A), as the maximum acceptable loss of azacitidine concentration.

This mean loss of 1.86%, corresponding to a loss of 4.52% relative to the initial theoretical concentration (25 mg/mL), occurred after 36 h of storage in all three Vidaza^®^ (25 mg/mL) lots stored in condition C, but in one lot occurred also within 48 h (Table 4).

To identify the limit of use of Vidaza^®^ (25 mg/mL) suspensions stored in condition C, the chemical stability of azacitidine in aqueous suspension was evaluated in triplicate on each considered Vidaza^®^ lot.

Three vials of each lot were reconstituted according to the SPC, stored in condition C, and analyzed until 96 h from preparation, under the chromatographic conditions described before.

By 48 h in the nine samples, a mean loss of azacitidine was 1.82% relative to the initial experimental concentration value (24.36 mg/mL), and 4.30% relative to the initial theoretical concentration (25 mg/mL) occurred, which was less than the loss of azacitidine identified as the maximum acceptable change of concentration (1.86%).

**Figure 14:**
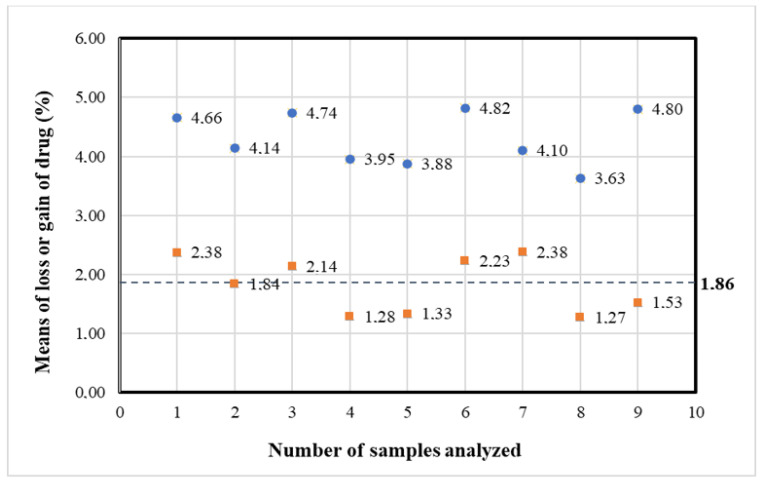
Percent loss of azacitidine relative to the initial experimental concentration value (red points), and the initial theoretical concentration (blue points) in nine samples at 48 h. The dotted line represents the maximum 1.86% loss (relative to the initial experimental concentration) identified as the maximum acceptable change of concentration.

Figure 14 representing the percentage loss of azacitidine relative to the baseline at 48 h shows a loss of Azacitidine at more than 1.86% in four of the nine samples (red points). However, a loss of less than 5% relative to the theoretical azacitidine concentration (25 mg/mL) occurred in all nine samples (blue points). Therefore, the Vidaza^®^ (25 mg/mL) suspensions could be used during the first 48 h from the reconstitution when stored according to condition C between two refrigerant gel packs.

This is a longer period than that suggested by the manufacturer (22 h) and can enable the preparation of the syringes in advance for administration on weekends, holidays, or in general when pharmacies may be closed. Furthermore, the withdrawal of the medicinal product into the polypropylene syringes immediately after the drug reconstitution is less difficult, because, over time, retention of the drug particles in the walls of the vial increase and, when a new dose must be prepared, there is the need to more vigorously shake the vial to favor a re-suspension and to make feasible the withdrawal of all of the suspension from the vial.

In our study, the influence of light on the in-use stability of the drug was not tested because, in our clinical practical conditions, all infusions and prefilled syringes were over-wrapped in light-protecting plastic bags. Moreover, Legeron et al. reported that the light caused no degradation or pH changes on Vidaza^®^ suspensions [12].

Regarding the choice of not also performing the UV-VIS spectrophotometry analysis to evaluate the chemical stability of the drug, it was due to the lack of suitability of this method to separate the intact drug from its DPs or excipients [1].

## 4. Materials and Methods

### 4.1. Chemicals and Reagents

Vidaza^®^ powder glass vials for injection suspension were purchased from Celgene (Milan, Italy).

Sterile water for injection was purchased from Fresenius Kabi Italia (Verona, Italy. Product number B315343).

Reusable refrigerant gel packs were purchased from VWR International (Leuven, Belgium. Product number 216-0192).

Azacitidine reference material, pharmaceutical grade, was purchased from Merck Life Science S.r.l. (Milan, Italy. Product number PHR1911).

Ammonium acetate, methanol, and acetonitrile (ACN) were purchased from Carlo Erba Reagents S.r.l. (Cornaredo, Milan, Italy); ultrapure water (Milli-Q, 18.2 MΩ) was obtained from a Milli-Q^®^ IQ Element purification (Merck KGaA, Darmstadt, Germany).

All chemicals were of analytical grade and were used without further purification.

HPLC eluents (Milli-Q water, methanol, and ACN) were of high-grade purity.

### 4.2. Design of the Stability Study

#### 4.2.1. Number and Analysis of Samples

The stability studies were performed on three different lots of Vidaza^®^ (lot 0F324A, expiration date 2024/05; lot 0H333A, expiration date 2024/07; lot 0I348A, expiration date 2024/08).

To study the intrinsic in-use stability of the product, the first experiment was performed on Vidaza^®^ stored in the original container (glass vial). The second one was carried out in the testing containers (3 mL BD Plastick Luer-Lock polypropylene syringes, product number 309658).

Complying with the ICH guidelines [27], the solvent used in clinical practice (sterile water for injection) was evaluated during the stability studies; the preparation procedure and syringes were those used in the daily practice.

To avoid potential microbial or particulate contamination, the Vidaza^®^ was reconstituted under aseptic conditions in a laminar flow hood.

Three different technicians reconstituted the Vidaza^®^, containing 100 mg of Azacitidine, with 4 mL of refrigerated (2–8 °C) sterile water for injection to form a 25 mg/mL suspension, according to SPC, at the centralized compounding unit of reconstitution and preparation of anticancer drugs of Policlinico di Modena.

Each Vidaza^®^ reconstituted lot was separated into three equal aliquots (1.3 mL), of which:

- One was stored refrigerated (2–8 °C) in the original container (according to SPC) placed in a light-protecting plastic bag (hereinafter condition A);
- One was transferred into a polypropylene syringe closed by a red cap, placed in a light-protecting plastic bag, and stored refrigerated (2–8 °C) (hereinafter condition B);
- One was transferred into a polypropylene syringe closed by a red cap, placed in a light-protecting plastic bag, and stored refrigerated (2–8 °C) between two refrigerant gel packs (hereinafter condition C).

To mimic the clinical practice, before analysis, samples were placed at room temperature for five minutes to reach the temperature of 22–28 °C, and vigorously shake to promote re-suspension and obtain a uniform suspension.

The sample stored in the original container (condition A) was analyzed at time zero and until 22 h from preparation; differently, other samples (conditions B and C) were analyzed at time zero and until 96 h.

Since the stability studies were performed on three different lots of Vidaza^®^, each time point was determined in triplicate.

#### 4.2.2. Temperature

Precise control of storage temperature was recorded throughout the study using a Marconi SPY U1 digital data logger (Giorgio Bormac s.r.l., Carpi, Italy), which automatically sampled (every minute) the temperature inside the two refrigerators where the samples were stored.

Moreover, to record the temperature of the Vidaza^®^ samples stored refrigerated (2–8 °C) between two refrigerant gel packs, a temperature data logger was placed into the polypropylene syringe in contact with the drug suspension. The devices had a 0.1 °C resolution with an accuracy of ±0.5 °C.

The data were then transferred to individual Excel spreadsheets (Excel 2000). The storage temperature of the samples was consistent with the practical conditions of the Vidaza^®^ suspension storage.

### 4.3. Stability Study

The stability study was performed following the guidelines for the practical stability studies of anticancer drugs from a European consensus conference, published by the French Society of Oncology Pharmacy (SFPO) [1].

The guidelines were based on ICH guidelines, particularly ICH Q1A (evaluation for stability data), ICH Q1A(R2) (stability testing of new drug substances and products), ICH Q2A (test on validation of analytical procedures), ICH Q1B (stability testing: photostability testing of new drug substances and products), Q3B (impurities in new drug products), Q5C (stability testing of biotechnological/biological product), European Pharmacopeia (Ph. Eur.), EMA guidelines, and the most relevant literature [3,25,27,29,30,31,32,33].

#### 4.3.1. Chemical Stability Analysis

The chemical stability of azacitidine in aqueous suspension was evaluated using the HPLC–UV system and the chromatographic conditions described later on.

The sample stored in the original container (condition A) was analyzed at time zero and times 4, 8, 12, and 22 h from preparation. Other samples (conditions B and C) were analyzed at time zero and times 4, 8, 12, 24, 36, 48, 54, 60, 64, 68, 72, and 96 h.

At each analyzing time, the Vidaza^®^ suspensions (25 mg/mL) were placed at room temperature for five minutes, then a 20 µL aliquot of each test sample was diluted with refrigerated (2–8 °C) sterile water for injection to a final concentration of 50 µg/mL to obtain a dilute solution for chromatographic analysis.

To calculate the azacitidine concentration (mg/mL) at each time point, the measured azacitidine % relative area was compared to the initial theoretical azacitidine % relative area (100%) that was correlated to the initial theoretical concentration of 25 mg/mL.

From the obtained azacitidine concentration (mg/mL), the percentage loss of azacitidine at each time point relative to the initial experimental concentration value was calculated.

The experiments were performed on triplicate samples (on three different lots of Vidaza^®^). The data were expressed as mean ± standard deviation (S.D.) and reported in a summary table.

##### Stability Limits

The mean loss of azacitidine occurring at 22 h from preparation in three different lots of Vidaza^®^, reconstituted and stored refrigerated (2–8 °C) in the original container according to SPC (condition A) was considered as the limit of chemical stability.

The chemical stability limit (as loss of azacitidine) was based on the remaining percentage of the initial experimental concentration value, which was calculated relative to the initial theoretical concentration (25 mg/mL).

##### HPLC–UV Analysis

HPLC analysis was performed on a Thermo Scientific Dionex Ultimate 3000 HPLC system (Thermo Scientific, Bremen, Germany) equipped with an LPG-3400SD pump, TCC-3000 column oven, and UV VWD-3100 detector.

According to the United States Pharmacopeia’s (USP) pending monograph for azacitidine [34] and certificate of analysis (CoA) of azacitidine reference material supplied by Merck Life Science S.r.l. (Milan, Italy), the HPLC analysis using a reversed-phase high-performance liquid chromatography (RP-HPLC; Ascentis Express C18, 150 mm × 4.6 mm, 2.7 μm; Merck Life Science S.r.l. (Milan, Italy) with a linear A-B gradient (0–4.8 min 0% B, 4.8–12 min 0% to 15% B, 12–15 min 15% B, 15–18 min 15% to 30% B, 18–24 min 30% to 50% B, 24–27 min 50% to 0% B, 27–33 min 0% B) at a flow rate of 0.8 mL/min and a total run time of 33 min was performed. Solvent A consisted of 1.54 g/mL ammonium acetate in water (0.02 M, pH 6.9 ± 0.1) and solvent B consisted of solvent A:methanol:acetonitrile (50:30:20).

UV absorbance was measured at 210 nm. The column temperature was kept at 30 °C. The injection volume was 20 μL.

The Chromeleon data system software (Version 7.2.8) was used for data acquisition and mathematical calculations.

The extensive validation of the analytical method was carried out according to ICH Q2(R1) guidelines [27] (see Supplementary Materials).

##### Forced Degradation Study

A forced degradation study was conducted out on one Vidaza^®^ preparation (25 mg/mL), to test the specificity and the suitability of the chromatographic method for use as a stability-indicating assay.

As a degradation test is designed to increase the rate of chemical degradation of the drug and determine the nature and chromatographic peaks of all DPs, the sample was exposed at 50 °C/43% RH for 12 h.

The used conditions were such to not obtain a drug degradation of more than 20% in order not induce the formation of DPs completely different from those observed in daily practice.

##### UHPLC-HRMS Analysis

To identify unknown impurities/degradation products formed during the proposed new storage paradigm of reconstituted azacitidine solutions, whose peaks were detected in the HPLC-UV trace, as to exclude their toxic potential, an ultra-high-performance liquid chromatography high-resolution mass spectrometry (UHPLC–HRMS) of samples stored in the condition C was performed.

Briefly, 2 µl of the 50 µg/mL solution were injected into a Thermo Scientific Dionex Ultimate 3000 UHPLC coupled to a Thermo Ultrahigh-resolution Q Exactive mass spectrometer (Thermo Scientific, Bremen, Germany). The column (Ascentis Express C18, 150 mm × 4.6 mm, 2.7 μm; Merck Life Science S.r.l. (Milan, Italy)), thermostatted at 30 °C, was equilibrated with 0.8 mL/min of 1.54 g/mL ammonium acetate in water (0.02 M, pH 6.9 ± 0.1) (solvent A); after 4.8 min from the sample injection, solvent B (solvent A:methanol:acetonitrile 50:30:20) was linearly increased from 0 to 15% in 7.2 min; B% was then kept constant for 3 min, then brought to 30% in 3 min. From minutes 18 to 24 B% was raised to 50% and brought back to 0% B for the reconditioning step. Each sample required a total run time of 31 min. Centroided MS and MS^2^ spectra were recorded in both positive and negative polarities from 100 to 1500 and 200 to 2000 *m*/*z* in full MS/dd-MS² (TOP2) mode, at a resolution of 70,000 and 17,500, respectively. The two most intense ions were selected for MS^2^ nitrogen-promoted collision-induced dissociation (NCE = 30). Precursor dynamic exclusion (15 s) and apex triggering (1 to 6 s) were set. The mass spectrometer was calibrated before the start of the analyses.

##### Infrared Spectroscopy Analysis

To assess the chemical changes to azacitidine structure over time, a Fourier transform infrared spectroscopy (FTIR) analysis was performed. The spectra were obtained with 32 scans in a Bruker Vertex 70 V FT-IR spectrometer (Bruker Optics, Ettlingen, Germany), equipped with a Hyperion microscope attachment.

The sample coated CaF_2_ slides were placed under the microscope objective and IR spectra were recorded in transmission mode from 4000 to 650 cm^−1^ at a spectral resolution of 4 cm^−1^. The spectra were collected using an attenuated total reflectance (ATR) diamond crystal (KRS-5 lens, Golden Gate model GS10542-K; Specac, Inc., Fort Washington, PA, USA) positioned within the optical bench of the spectrometer.

The sample stored in the original container (condition A) was analyzed at time zero and 22 h from preparation. Other samples (conditions B and C) were analyzed at time zero and times 24, 48, 72, and 96 h. The experiments were performed on three different lots of Vidaza^®^.

##### pH Determination

The determination of the pH value was performed with an electrochemical method using one micro-electrode and a millivoltmeter (pH meter) from Thermo Scientific™ Orion™ Dual Star.

The sample stored in the original container (condition A) was analyzed at time zero and 22 h from preparation. Other samples (conditions B and C) were analyzed at time zero and times 24, 48, 72, and 96 h. The experiments were performed on three different lots of Vidaza^®^.

#### 4.3.2. Physical Stability Analysis

##### Visual Examination

Whenever samples were taken for analysis, vials and syringes were visually checked to assay the change in the initial color or appearance or particulate matter of the suspension.

##### Subvisual Examination

To evaluate the changes in terms of shape, size, and number of particles, as well as to examine any sign of physical instability, such as aggregation or particle precipitation, microscopic observation and the particle counter were performed.

Microscopic observation was performed following the 2.9.37 current test of European Pharmacopeia [35].

Since in the hospital laboratory, the method light obstruction [36] or turbidimetry [37] based was not available, the size and quantity of particles were evaluated by image-based cytometry [38], which is known to provide comparable data to traditional flow cytometry [39].

The sample stored in the original container (condition A) was analyzed at time zero and 22 h from preparation. Other samples (conditions B and C) were analyzed at time zero and times 24, 48, 72, and 96 h. The experiments were performed on three different lots of Vidaza^®^.

##### Microscopic Observation

The evaluation of crystal morphology was performed on microscopy slides loaded with samples and acquired using a computer (equipped with uEye UI-1460LE-C features a 1/2 inch CMOS sensor with a 2048 × 1536 pixel resolution color sensor) and the program MultiScan v.8.08 Computer Scanning System. The computer was connected to an Olympus BX 40 microscope with a 10× objective (NA 0.25).

##### Particle and Size Counting

The quantity and the size of azacitidine particles were evaluated with Tali^®^ Image-Based cytometer. For the assay, the Tali^®^ cellular analysis slides were used. The slide holds the sample in two separate, enclosed chambers. In each chamber, 25 μL of the sample were loaded. The Tali^®^ Image-Based cytometer captures a series of images (i.e., fields of view) of the sample in the chamber and then analyzes them using algorithms specifically designed to determine total particle counts in a range between 0–60 µm and calculates their concentrations in 1 mL.

#### 4.3.3. Microbiological Stability Analysis

Classically, it is considered that many anticancer drugs do not facilitate bacterial growth. Moreover, thanks to the application of good hospital pharmacy manufacturing practice rules, sterile conditions were guaranteed during the manufacturing process, preventing bacterial contamination. Nevertheless, since the maintenance of the sterility in the final container also depends on the nature of the container and the storage conditions, the sterility assay was performed.

##### Sterility Assay

The study took place at the Biochem Microbiology Laboratory (Zola Pedrosa, Bologna, Italy).

The sterility assay was performed in triplicate for each storage condition at 22 (condition A) and 96 (conditions B and C) hours from preparation, respectively.

The methodology of the test followed the 2.6.1 current test of the European Pharmacopoeia [40].

The seeding was carried out under a vertical laminar air-flow hood in aseptic conditions and the containers were decontaminated externally with 70° of alcohol.

Then, 1.5 mL of each sample was transferred directly into the thioglycollate medium for the detection of aerobic and anaerobic micro-organisms and into the tryptone soya broth medium for the detection of fungi. Tubes were then incubated for 14 days at 22 ± 2 °C and 32 ± 2 °C, respectively, and observed at 4 and 14 days of incubation. The results were considered satisfactory if no evidence of microbial growth is found. Appropriate negative controls were included.

## 5. Conclusions

Our results demonstrated that the refrigeration (2–8 °C) associated with the use of refrigerant gel packs efficiently delays degradation of azacitidine and makes it possible to extend the stability of Vidaza^®^ (25 mg/mL) suspensions.

The reconstitution of the Vidaza^®^ according to SPC and the immediate transfer of the suspension into polypropylene syringes stored refrigerated (2–8 °C) between two refrigerant gel packs makes it possible to prepare in advance Vidaza^®^ (25 mg/mL) syringes which can be used up to 48 h from the preparation. Indeed, each preparation kept its physicochemical and microbiological properties from the beginning of the preparation until 48 h from the preparation.

Furthermore, the withdrawal of the medicinal product into the polypropylene syringes immediately after the drug reconstitution makes it feasible to more easily withdraw the suspension from the vial.

We advise applying the shortest demonstrated in-use stability value (i.e., 36 h) if the suspension is allowed to room temperature for a time longer than five minutes, for example, for re-using the drug when the administration is canceled or postponed.

This new storage procedure was validated under our specific routine clinical operating conditions and materials (i.e., reusable refrigerant gel packs VWR International, product number 216-0192). Each compounding unit that wants to use this new storage procedure should verify it (e.g, by analysis HPLC) to make sure to have implemented the method properly according to its specific routine clinical operating conditions and should then define it in local standardized procedures.

Nevertheless, it is important to be aware of the legal issues related to the administration of drugs over the limits of use declared by the manufacturer.

## Figures and Tables

**Table 1:** Chemical stability of Vidaza^®^ (25 mg/mL) stored at 2–8 °C in the original container (condition A), in a polypropylene syringe (condition B), and in a polypropylene syringe placed between two refrigerant gel packs (condition C).

Storage Time (h)	Vidaza^®^ (25 mg/mL) Stored into Condition A		Vidaza^®^ (25 mg/mL) Stored into Condition B		Vidaza^®^ (25 mg/mL) Stored into Condition C	
	Mean ± SD Drug Concentration (mg/mL) ^b^	Mean Loss or Gain of Drug (%) ^c^	Mean ± SD Drug Concentration (mg/mL) ^b^	Mean Loss or Gain of Drug (%) ^c^	Mean ± SD Drug Concentration (mg/mL) ^b^	Mean Loss or Gain of Drug (%) ^c^
**0**	24.33 ± 0.11	- ^d^	24.33 ± 0.11	- ^d^	24.33 ± 0.11	- ^d^
**4**	24.25 ± 0.11	−0.30	24.22 ± 0.03	−0.45	24.35 ± 0.09	0.08
**8**	24.18 ± 0.14	−0.61	24.16 ± 0.04	−0.69	24.30 ± 0.05	−0.12
**12**	24.06 ± 0.19	−1.09	24.10 ± 0.04	−0.95	24.14 ± 0.07	−0.79
**22**	23.87 ± 0.09	−1.86				
**24**			23.91 ± 0.03	−1.73	24.07 ± 0.04	−1.07
**36**			23.83 ± 0.10	−2.04	23.98 ± 0.04	−1.46
**48**			23.70 ± 0.08	−2.57	23.81 ± 0.08	−2.12
**54**			23.62 ± 0.04	−2.94	23.77 ± 0.05	−2.29
**60**			23.59 ± 0.10	−3.05	23.66 ± 0.08	−2.74
**64**			23.50 ± 0.13	−3.41	23.62 ± 0.07	−2.93
**68**			23.39 ± 0.14	−3.85	23.50 ± 0.06	−3.43
**72**			23.39 ± 0.07	−3.87	23.55 ± 0.05	−3.21
**96**			23.17 ± 0.06	−4.77	23.23 ± 0.08	−4.52

^b^ All assays were performed in triplicate. ^c^ Cumulative change from t_0_. ^d^ Not applicable.

**Table 2:** pH of Vidaza^®^ (25 mg/mL) stored at 2–8 °C in the original container (condition A), in a polypropylene syringe (condition B), and in a polypropylene syringe placed between two refrigerant gel packs (condition C).

Storage Time (h)	Vidaza^®^ (25 mg/mL) Stored in Condition A	Vidaza^®^ (25 mg/mL) Stored in Condition B	Vidaza^®^ (25 mg/mL) Stored in Condition C
	Mean ± SD pH	Mean ± SD pH	Mean ± SD pH
**0**	6.81 ± 0.06	6.81 ± 0.06	6.81 ± 0.06
**22**	7.25 ± 0.08		
**24**		7.18 ± 0.10	6.86 ± 0.06
**48**		7.36 ± 0.11	7.22 ± 0.02
**72**		7.34 ± 0.11	7.27 ± 0.03
**96**		7.40 ± 0.10	7.27 ± 0.03

**Table 3:** Results of particles size and number by Tali^®^ Image-Based Cytometer.

Storage Time (h)	Vidaza^®^ (25 mg/mL) Stored in Condition A		Vidaza^®^ (25 mg/mL) Stored in Condition B		Vidaza^®^ (25 mg/mL) Stored in Condition C	
	Average Particles Size (µm)	# of Particles Counted	Average Particles Size (µm)	# of Particles Counted	Average Particles Size (µm)	# of Particles Counted
**0**	18 ± 2.0	1182 ± 52	18 ± 2	1182 ± 52	18 ± 2	1182 ± 52
**22**	21 ± 1.5	2466 ± 86				
**24**			21 ± 0.6	1184 ± 102	21 ± 2.0	475 ± 27
**48**			22 ± 2.0	1371 ± 42	23 ± 2.0	997 ± 39
**72**			22 ± 3.8	1552 ± 77	24 ± 0.5	2116 ± 165
**96**			24 ± 2.5	3025 ± 143	24 ± 0.0	2435 ± 103

**Table 4:** Loss or gain of Azacitidine in three Vidaza^®^ (25 mg/mL) preparations stored in conditions A and C at each time study.

Storage Time (h)	Vidaza^®^ (25 mg/mL) Stored in Condition A				Vidaza^®^ (25 mg/mL) Stored in Condition C			
	Loss or Gain of Drug (%) ^c^ lot 0F324A	Loss or Gain of Drug (%) ^c^ lot 0H333A	Loss or Gain of Drug (%) ^c^ lot 0I348A	Mean Loss or Gain of Drug (%)^c^	Loss or Gain of Drug (%) ^c^ lot 0F324A	Loss or Gain of Drug (%) ^c^ lot 0H333A	Loss or Gain of Drug (%) ^c^ lot 0I348A	Mean Loss or Gain of Drug (%)^c^
**0**	- ^d^	- ^d^	- ^d^		- ^d^	- ^d^	- ^d^	
**4**	0.06	−0.41	−0.57	−0.30	0.24	0.12	−0.11	0.08
**8**	0.09	−0.89	−1.02	−0.61	−0.01	−0.23	−0.12	−0.12
**12**	−0.24	−1.25	−1.77	−1.09	−0.99	−0.57	−0.79	−0.79
**22**	−1.81	−1.92	−1.84	**−1.86**				
**24**					−1.21	−1.31	−0.70	−1.07
**36**					**−1.71**	**−1.36**	**−1.29**	−1.46
**48**					−2.38	**−1.84**	−2.14	−2.12
**54**					−2.47	−2.14	−2.25	−2.29
**60**					−3.07	−2.49	−2.65	−2.74
**64**					−3.35	−2.77	−2.67	−2.93
**68**					−3.34	−3.47	−3.47	−3.43
**72**					−3.54	−3.22	−2.88	−3.21
**96**					−5.15	−4.77	−4.40	−4.77

^c^ Cumulative change from t_0_. ^d^ Not applicable.

## Data Availability

Data is contained within the article and supplementary materials.

## Supplementary Materials

The following are available online at https://www.mdpi.com/article/10.3390/ph14090943/s1, Table S1: Results of repeatability (1 day) and intermediate precision (1–3 days) from validation studies for Azacitidine, Table S2: Results of accuracy from validation studies for Azacitidine, Table S3: Robustness data, Figure S1: Standard Calibration Curve of Azacitidine, Figure S2: ESI+ HRMS spectrum of RGU—tautomer 1, Figure S3: ESI+ HRMS spectrum of RGU—tautomer 2, Figure S4: ESI+ HRMS spectrum of RGU—tautomer 3, Figure S5: ESI+ HRMS spectrum of RGU-CHO, Figure S6: ESI+ HRMS spectrum of the hydrated form of Azacitidine or the RGU-CHO tautomer, Figure S7: ESI+ HRMS spectrum of Azacitidine.
