# Supplementary material for: Development and Validation of a New Storage Procedure to Extend the In-Use Stability of Azacitidine in Pharmaceutical Formulations
Source: Pharmaceuticals (Basel). 2021 Sep 21;14(9):943. doi: 10.3390/ph14090943 (PMC8470010; doi:10.3390/ph14090943)
Supplement: Supplementary file 1 [file pharmaceuticals-14-00943-s001.zip › pharmaceuticals-1308002-supplementary.pdf]

pharmaceuticals

Supplementary Materials

Development and Validation of a New Storage Procedure to Extend the In-Use Stability of Azacitidine in Pharmaceutical Formulations

Antonella Iudicello, Filippo Genovese, Valentina Strusi, Massimo Dominici and Barbara Ruozi

Citation: Iudicello, A.; Genovese, F.; Strusi, V.; Dominici, M.; Ruozi, B. Development and Validation of a New Storage Procedure to Extend the In-Use Stability of Azacitidine in Pharmaceutical Formulations. *Pharmaceuticals* **2021**, *14*, 943. https://doi.org/10.3390/ph14090943

Academic Editors: María Ángeles Peña Fernández and Serge Mordon

Received: 5 July 2021
Accepted: 9 September 2021
Published: 21 September 2021

Publisher's Note: MDPI stays neutral with regard to jurisdictional claims in published maps and institutional affiliations.

Copyright: © 2021 by the authors. Licensee MDPI, Basel, Switzerland. This article is an open access article distributed under the terms and conditions of the Creative Commons Attribution (CC BY) license (http://creativecommons.org/licenses/by/4.0/).

S1. Validation of the HPLC-UV assay method

The USP pending monograph for azacididine has not yet been released for publication. Furthermore, analytical methods described in Pharmacopoeia are primarily developed to find impurities arising from the synthesis in the corresponding raw chemical and not to evaluate DPs being formed in thepractical use of the drug.

Therefore, the extensive validation of the analytical method, described in the USP pending monograph for the impurity analysis of azacitidine, was carried out according to ICH Q2(R1) guidelines [1].

S1.1. Materials and methods

To validate the analytical method azacitidine reference material (purecompound) was used.

The parameters assessed were: specificity, linearity, range, detection limit (LOD), quantification limit (LOQ), precision, accuracy, and robustness.

Azacitidine stock solution was prepared from the entire contents of one vial (i.e., 300 mg of azacitidine reference material lyophilized powder) reconstituted with 3000 mL of refrigerated (2–8 °C) sterile water for injection to produce a final concentration of 100 μg/mL.

S1.1.1. Specificity

Specificity is the ability of the chromatographic method to assess unequivocally the analyte in the presence of components, which may be expected to be present. Typically, these might include vehicles, diluents (i.e. water for injection) or DPs being formed in the practical use of the drug, and excipients (i.e. mannitol).

The specificity of the method was determined by analyzing: the diluent (water for injection), a 50 μg/mL azacitidine standard solution freshly prepared, and one sample of Vidaza® (50 μg/mL) subjected to heat stress to induce the complete formation of DPs (see "forced degradation study" paragraph).

No interference due to mannitol was evaluated since the mannitol detection needs an HPLC with pulsed amperometric detection.

All chromatograms were examined to determine if azacitidine peak and diluent or DPs peaks co-eluted.

The resolution (Rs) between different peaks of interest was calculatedusing the following equation:

$$Rs = 1.18 [(t_2 - t_1)/(W_2 + W_1)] \quad (1)$$

where t_2 and t_1 are the retention times of analytes, and W_2 and W_1 are the peak widths at half height.

S1.1.2. Linearity and range

Linearity of the method was established using five standard solutions from diluting the azacitidine stock solution (100 μg/mL) with refrigerated (2–8 °C) sterile water for injection to produce concentrations of 25, 37.5, 50, 62.5, and75 μg/mL, corresponding to concentrations of ± 50% of the working solution (50 μg/mL).

Reconstituted azacitidine in an aqueous medium is known to be unstable.So, to minimize the azacitidine degradation during the analytical process, the stock solution and the standard solutions were immediately aliquoted (1 mL and 800 μL, respectively) in 1–0.5 mL Eppendorf tubes and frozen at -25 °C [2- 5].

Each aliquot was thawed before analysis. Three replicates of each concentration were injected.

The data of peak area versus concentration was subjected to least-square regression analysis.

S1.1.3. Detection limit and quantitation limit determination

LOD and LOQ values were determined according to equations:

$$LOD_{calibration} = 3.3 \times (\sigma/s) \quad (2)$$

$$LOQ_{calibration} = 10 \times (\sigma/s) \quad (3)$$

where σ is the standard deviation of the response or standard deviation of y-intercepts, and s is the slope of the standard calibration curve.

S1.1.4. Precision

The precision was measured as repeatability (or Intra-day precision) and intermediate precision (or Inter-day precision).

Intra-day precision was assessed using three concentrations: 25, 50, and 75μg/mL (low, medium, and high concentrations on the standard calibration curve), and through the calculation of the coefficient of variation (CV) (or Relative Standard Deviation value (RSD) of the peak areas, as defined by ICH Q2(R1) guidelines:

$$RSD = (SD/\text{Mean}) \times 100 \quad (4)$$

where SD is the standard deviation [1].

Three replicates of each concentration were injected under the sameoperating conditions over a short interval of time.

Inter-day precision was assessed by injecting three replicate of 25, 50, and 75 μg/mL concentrations (low, medium, and high concentrations on the standard calibration curve) on three consecutive days, and through the variance calculation (ANOVA), which allows, in turn, for Fisher value (F)calculation.

In repeatability and intermediate precision, the coefficient of variationshould not exceed 5%.

S1.1.5. Accuracy

The accuracy of the analytical procedure expresses the degree of the closeness of the obtained results with the theoretical values. The accuracy ofthe method was evaluated at three different concentrations namely, 50%, 100%,and 150% of the target assay concentration, and was expressed as percent deviation assessed on the deviation between the known concentration valuesof injected standards and the obtained concentration values from the standard calibration curve.

The accuracy of the analyte should be within the range of 98–102%.

S1.1.6. Robustness

The robustness of an analytical method can be measured by its capacity to remain unaffected by small but deliberate changes in the practical chromatography conditions.

The robustness of the method was assessed by making deliberate variations in the HPLC pump flow rate (± 10%), column compartment temperature (± 4°C), and Active Back Pressure Regulator (ABPR).

S1.2. Results

S1.2.1. Specificity

No interference was observed at the average RT of the azacitidine peak (RT ~ 4.3 min): the diluent and the obtain degradation by-products peaks were separated from the azacitidine peak (Figure 4).

The resolution (Rs) between different peaks and the azacitidine peak was always greater than 1.5 indicating the specificity of the method for azacitidine: the contents of the active ingredient, DPs, and other components can be accurately measured without interference.

S1.2.2. Linearity and range

The linearity of the method was confirmed by preparing a standard calibration curve for the analytical range of 25–75 μg/mL.

The linear regression data from the calibration plot were indicative of an excellent linear relationship between the analyte peak area and concentration over the range specified above (Figure S1). A correlation (r^2) greater than 0.999 was found. The regression intercepts were not significantly different from zero (y = 0.1679x–0.1941).

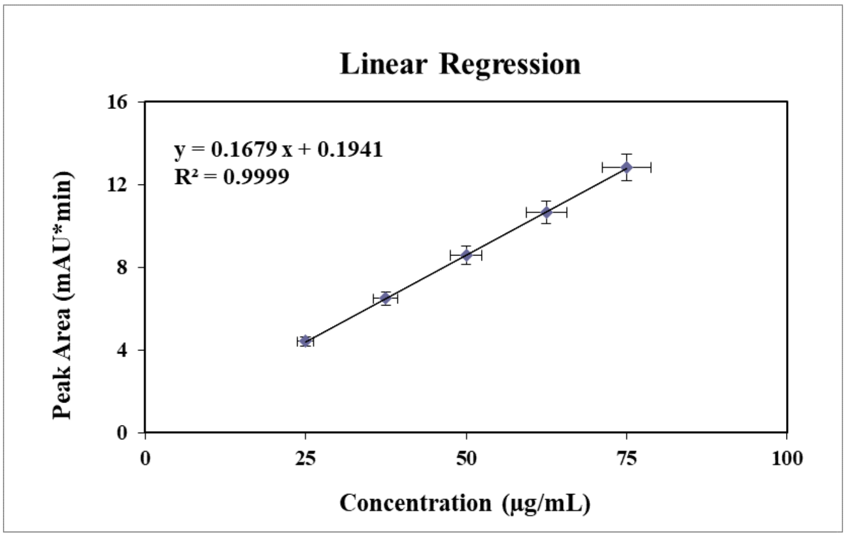

Figure S1. Standard Calibration Curve of azacitidine.

The analytical range was 50-150% w/w.

S1.2.3. Detection Limit and Quantitation Limit

Limit of detection and limit of quantitation for azacitidine were evaluated to determine the sensitivity of the analytical method.

The obtained limit of quantitation based on 10 × (σ/s) was 0.86 μg/mL. Theobtained limit of detection based on 3.3 × (σ/s) was 0.29 μg/mL.

S1.2.4. Precision

The repeatability of the assay was determined by carrying out the analysis on three different concentrations. The standard deviation was calculated from the obtained peak areas. A % RSD less than 1% with a standard deviation less than 0.1 was found for each concentration, compared with acceptance criteria of 5%. This shows that the method is precise according to ICH guidelines [1]. The results are summarized in Table S1.

The analysis of variance (ANOVA) allowed evaluating the intermediate precision with the post-Fischer test using a significant level of $\alpha = 0.05$ (95% of confidence interval). As shown in Table S1, the value obtained for the $F_{(calculated)}$ is lower than the $F_{(tabulated)}$ ($F_{(tabulated)} = 5.140$), indicating no significant difference between the values obtained (peak area/corresponding concentration) from the calibration curves ($p > 0.05$).

Table S1. Results of repeatability (1 day) and intermediate precision (1–3 days) from validation studies for azacitidine.

		Solutions 25 µg/mL Peak Area (mAU*min)		Solutions 50 µg/mL Peak Area (mAU*min)		Solutions 75 µg/mL Peak Area (mAU*min)	
Day 1	Injection-1	4.4159		8.6616		12.9110	
	Injection-2	4.4157		8.5562		12.8154	
	Injection-3	4.3867		8.5241		12.7243	
	Mean	4.4061		8.5806		12.8169	
	SD	0.02		0.07		0.09	
	% RSD	0.38		0.84		0.73	
Day 2	Injection-1	4.5273		8.7490		13.0520	
	Injection-2	4.4096		8.6032		12.9083	
	Injection-3	4.4024		8.4962		12.8194	
	Mean	4.4464		8.6161		12.9266	
	SD	0.07		0.13		0.12	
	% RSD	1.58		1.47		0.91	
Day 3	Injection-1	4.4784		8.2392		12.5512	
	Injection-2	4.1731		8.7352		12.5146	
	Injection-3	4.1803		8.2221		12.7836	
	Mean	4.2773		8.3988		12.6165	
	SD	0.17		0.29		0.15	
	% RSD	4.07		3.47		1.16	
		Between groups	Within groups	Between groups	Within groups	Between groups	Within groups
SS-Sum of squares		0.0468	0.0711	0.08	0.21	0.15	0.09
DF-degrees of freedom		2	6	2	6	2	6
MS-Mean square		0.0234	0.0119	0.04	0.04	0.07	0.01
Fisher F		1.976		1.151		5.083	
p-value		0.219		0.377		0.051	

S1.2.5. Accuracy

For the correct analysis of an analyte, the method must be accurate, there must be a correlation between the obtained concentration values from thestandard calibration curve and the known concentration values of the injected standard. The results show that the obtained concentration value was within the range of 98–102% of the known concentration value. Thus, the chromatographic method presented accuracy (Table S2).

Table S2. Results of accuracy from validation studies for azacitidine.

	Solutions		
	**25 μg/mL**	**50 μg/mL**	**75 μg/mL**
**Injection-1**	100.6	100.9	101.0
**Injection-2**	100.6	99.6	100.2
**Injection-3**	99.9	99.2	99.5
**Mean**	**100.4**	**99.9**	**100.3**
**SD**	**0.4**	**0.8**	**0.7**

S1.2.6. Robustness

None of the alterations (variations in the HPLC pump flow rate (± 10%), column compartment temperature (± 4°C), and ABPR) caused a significant change (greater than ± 10%) in RT peak, peak area, and resolution between peaks of interest. Thus, the chromatographic method presented robustness.The various altered conditions are shown in Table S3.

Table S3. Robustness data.

**Parameters**		**RT of azacitidine (minutes)**	**Peak area of azacitidine 50 μg/mL**	**Resolution between azacitidine and RGU-CHO peaks**	**Resolution between azacitidine and RGU peaks**
**Control (no change)**		4.368	8.603	13.06	17.73
**Flux (mL.min−1)**	0.9	4.213	8.524	12.87	17.60
**Flux (mL.min−1)**	0.7	4.407	8.749	13.04	17.72
**Temperature (˚C)**	25°C	4.740	8.523	13.23	18.03
**Active back pressure regulator**	(+) 250	4.520	8.481	12.62	17.53

S2. High Resolution mass spectra of azacitidine and itsdegradation products

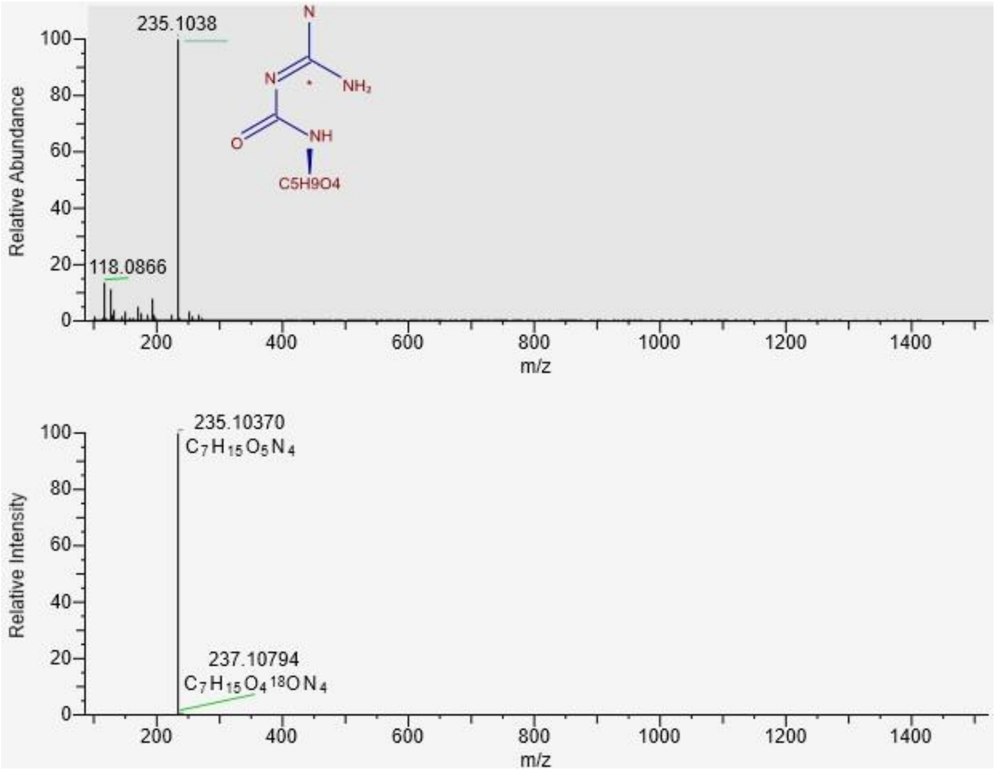

Figure S2. ESI+ HRMS spectrum of RGU – tautomer 1.

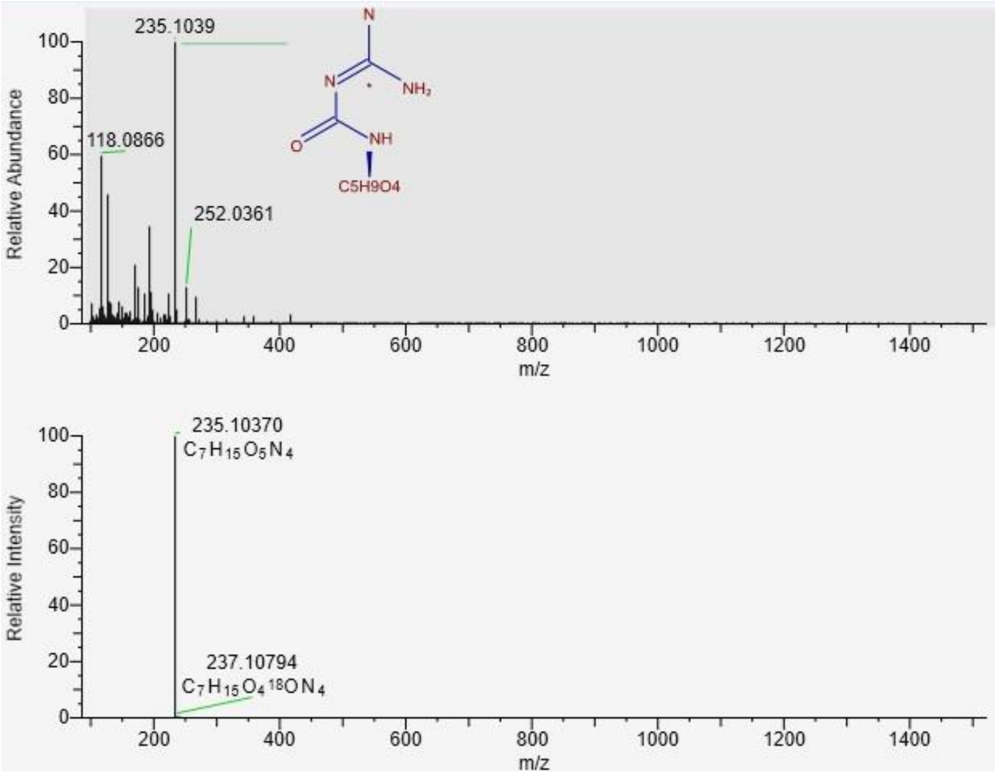

Figure S3. ESI+ HRMS spectrum of RGU–tautomer 2.

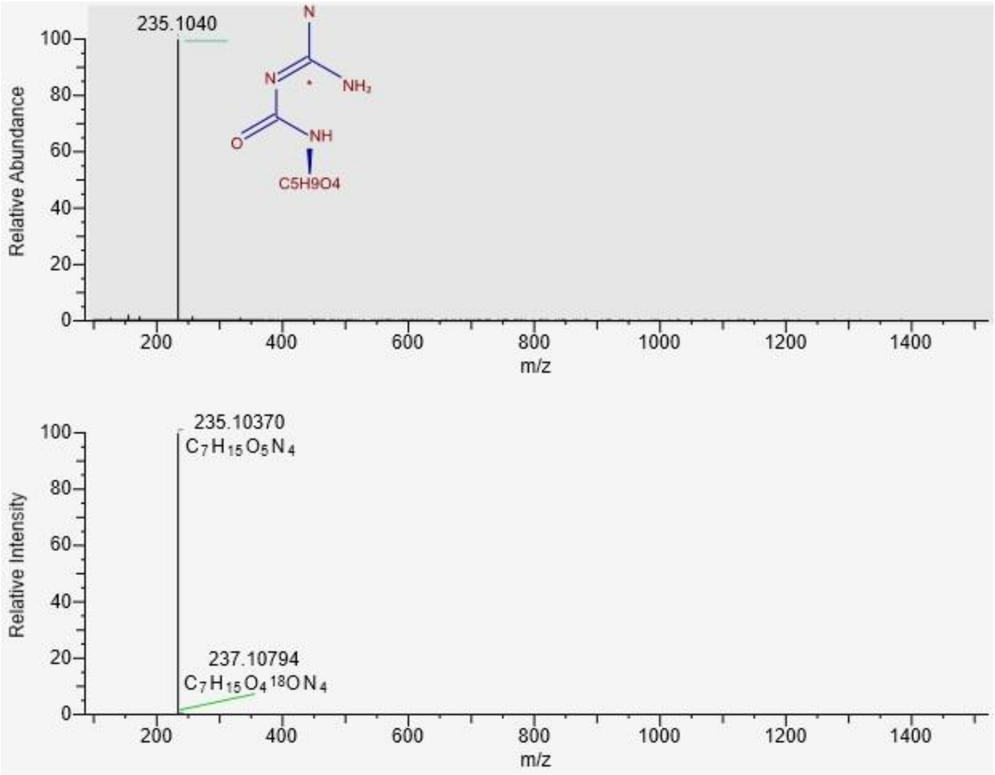

Figure S4. ESI+ HRMS spectrum of RGU–tautomer 3.

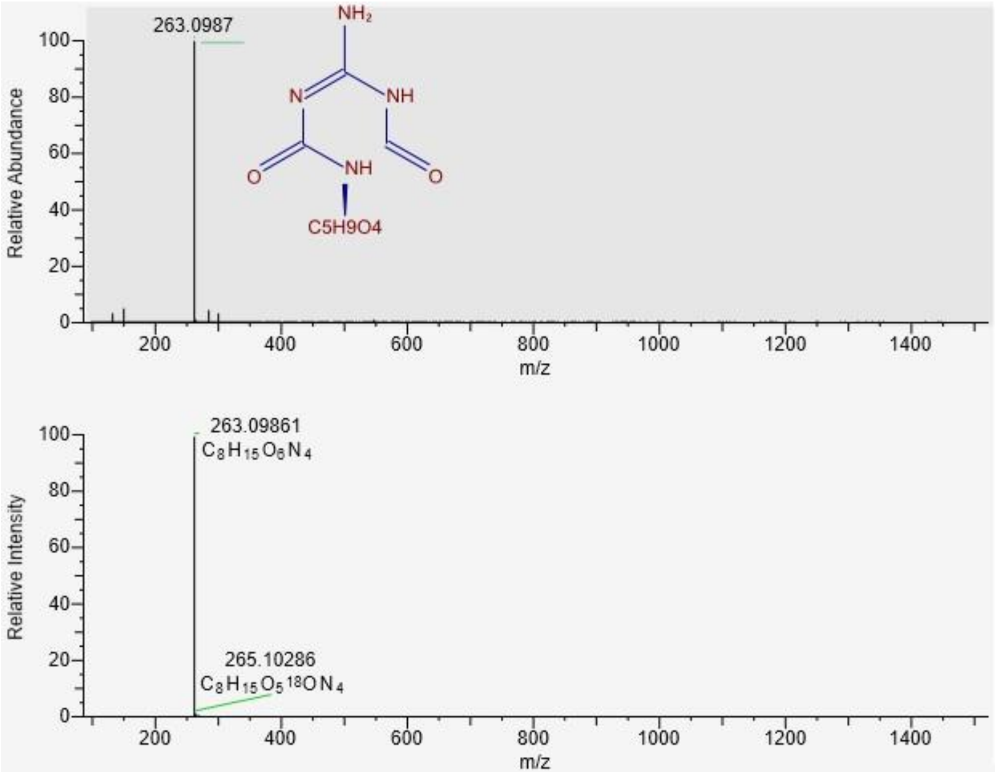

Figure S5. ESI+ HRMS spectrum of RGU-CHO.

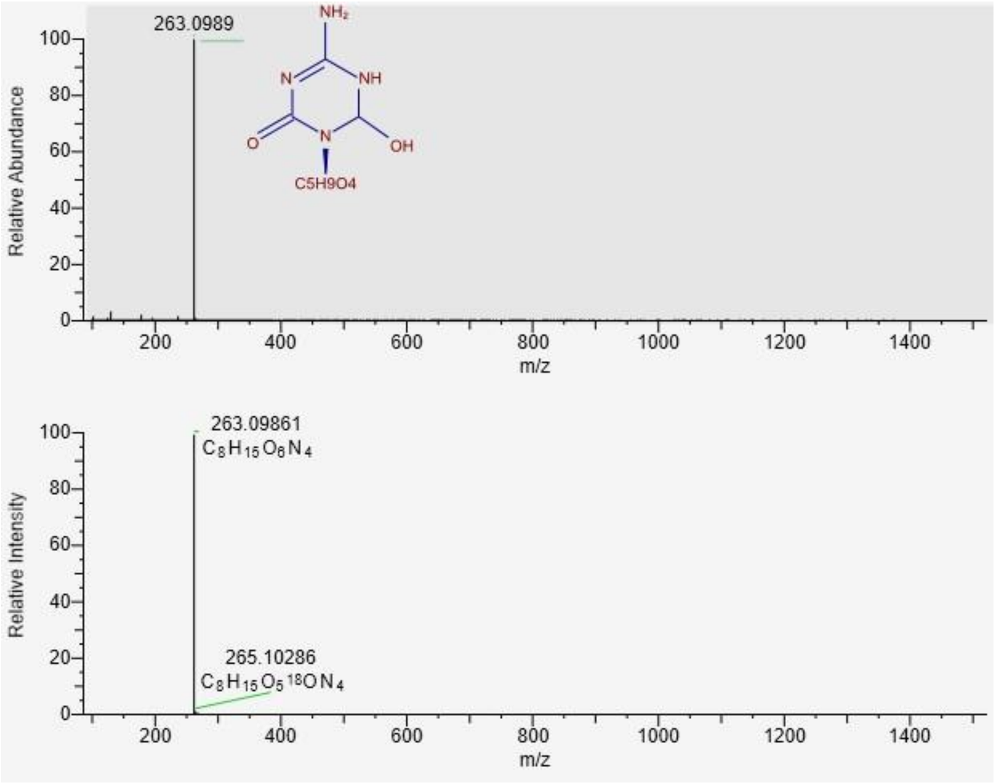

Figure S6. ESI+ HRMS spectrum of the hydrated form of azacitidine or the RGU-CHOtautomer.

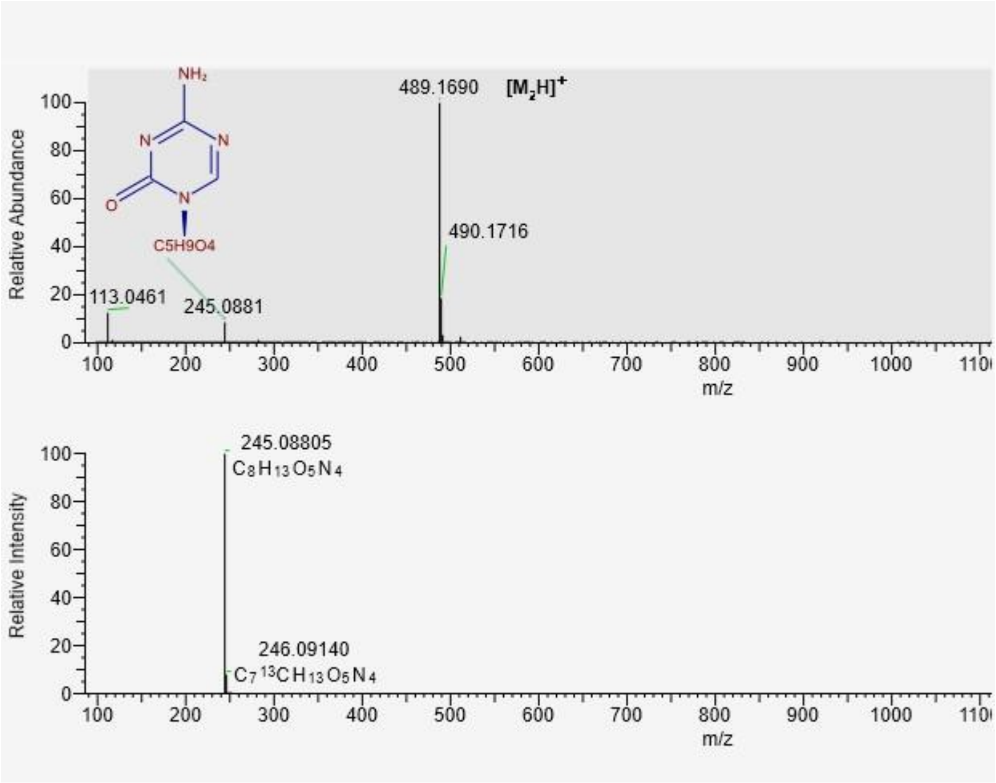

Figure S7. ESI+ HRMS spectrum of azacitidine.

References

[1] International Conference of Harmonization of Technical Requirements for Pharmaceuticals for Human Use.Guidelines for stability 2011. Available at: https://www.ich.org/products/guidelines. (Accessed May 2021).

[2] Hartigh, J.D., Brandenburg, H.C.R., Vermeij, P., 1989. Stability of azacitidine in lactated Ringer's injection frozen inpolypropylene syringes. American Journal of Hospital Pharmacy. 46 (12), 2500-2505.

[3] Walker, S.E., Charbonneau, L.F., Law, S., Earle, C., 2012. Stability of azacitidine in sterile water for injection. Can J Hosp Pharm. 65 (5), 352-359.

[4] Legeron, R., Xuereb, F., Djabarouti, S., Saux, M.-C., Breilh, D., 2013. Chemical stability of azacitidine suspensions forinjection after cold-chain reconstitution of powder and storage. Am J Health Syst Pharm. 70 (23), 2137-2142.

[5] Balouzet, C., Chanat, C., Jobard, M., Brandely-Piat M.-L., Chast, F., 2017. Stability of 25 mg/mL AzacitidineSuspensions Kept in Fridge after Freezing. Pharm. Technol. Hosp. Pharm. 2 (1), 11–16.
